# *In Vivo* IFN-γ Secretion by NK Cells in Response to *Salmonella* Typhimurium Requires NLRC4 Inflammasomes

**DOI:** 10.1371/journal.pone.0097418

**Published:** 2014-05-14

**Authors:** Andreas Kupz, Roy Curtiss, Sammy Bedoui, Richard A. Strugnell

**Affiliations:** 1 Department of Microbiology and Immunology, The University of Melbourne, Parkville, Victoria, Australia; 2 Center of Infectious Diseases and Vaccinology, Arizona State University, Tempe, Arizona, United States of America; 3 Max Planck Institute for Infection Biology, Berlin, Germany; 4 Queensland Tropical Health Alliance Research Laboratory, James Cook University, Cairns, Queensland, Australia; University of São Paulo, Brazil

## Abstract

Natural killer (NK) cells are a critical part of the innate immune defense against viral infections and for the control of tumors. Much less is known about how NK cells contribute to anti-bacterial immunity. NK cell-produced interferon gamma (IFN-γ) contributes to the control of early exponential replication of bacterial pathogens, however the regulation of these events remains poorly resolved. Using a mouse model of invasive Salmonellosis, here we report that the activation of the intracellular danger sensor NLRC4 by *Salmonella*-derived flagellin within CD11c^+^ cells regulates early IFN-γ secretion by NK cells through the provision of interleukin 18 (IL-18), independently of Toll-like receptor (TLR)-signaling. Although IL18-signalling deficient NK cells improved host protection during *S*. Typhimurium infection, this increased resistance was inferior to that provided by wild-type NK cells. These findings suggest that although NLRC4 inflammasome-driven secretion of IL18 serves as a potent activator of NK cell mediated IFN-γ secretion, IL18-independent NK cell-mediated mechanisms of IFN-γ secretion contribute to *in vivo* control of *Salmonella* replication.

## Introduction

The pathogen *Salmonella enterica* includes serovars that are the cause of typhoid fever, gastroenteritis and non-typhoidal bacteraemia in immunocompromised humans, conditions with considerable morbidity and mortality [1]. Although the mechanisms that lead to immunity against *S. enterica* are not fully understood, it is well established that the cytokine interferon-γ (IFN-γ) plays a critical role in the initial control of experimental and clinical Salmonellosis [2]. Understanding the cellular mechanisms that drive IFN-γ production during Salmonellosis is therefore essential for the development of novel treatment options and improved vaccines.

Using a murine model for invasive Salmonellosis, we have recently reported on the antigen-independent production of IFN-γ by memory CD8^+^ T cells through a complex *in vivo* mechanism that involves intracellular sensing of flagellin by NLRC4 inflammasomes, which is an important contributor to protection against invasive murine Salmonellosis [3]. Together with our recent demonstration that NK cells can also serve as key producers of host-protective IFN-γ during invasive Salmonellosis [4], these findings suggest that there may be functional overlap between NK cells and memory CD8^+^ T cells in the context of innate control of invasive Salmonellosis. Furthermore, studies have recently demonstrated the importance of antigen-independent IFN-γ production by memory CD8^+^ T cells and NK cells in other viral and bacterial infection models [5]. In order to potentially exploit IFN-γ production, for example, in settings where adaptive immune responses are absent or impaired, such as during severe *S*. Typhimurium infections in immunocompromised individuals [6] the present study was therefore designed to resolve the mechanism of NK cell-dependent early IFN-γ production during *Salmonella* infection.

## Results and Discussion

### NK cells rapidly produce IFN-γ in response to *S*. Typhimurium-derived flagellin

**Figure 1:**
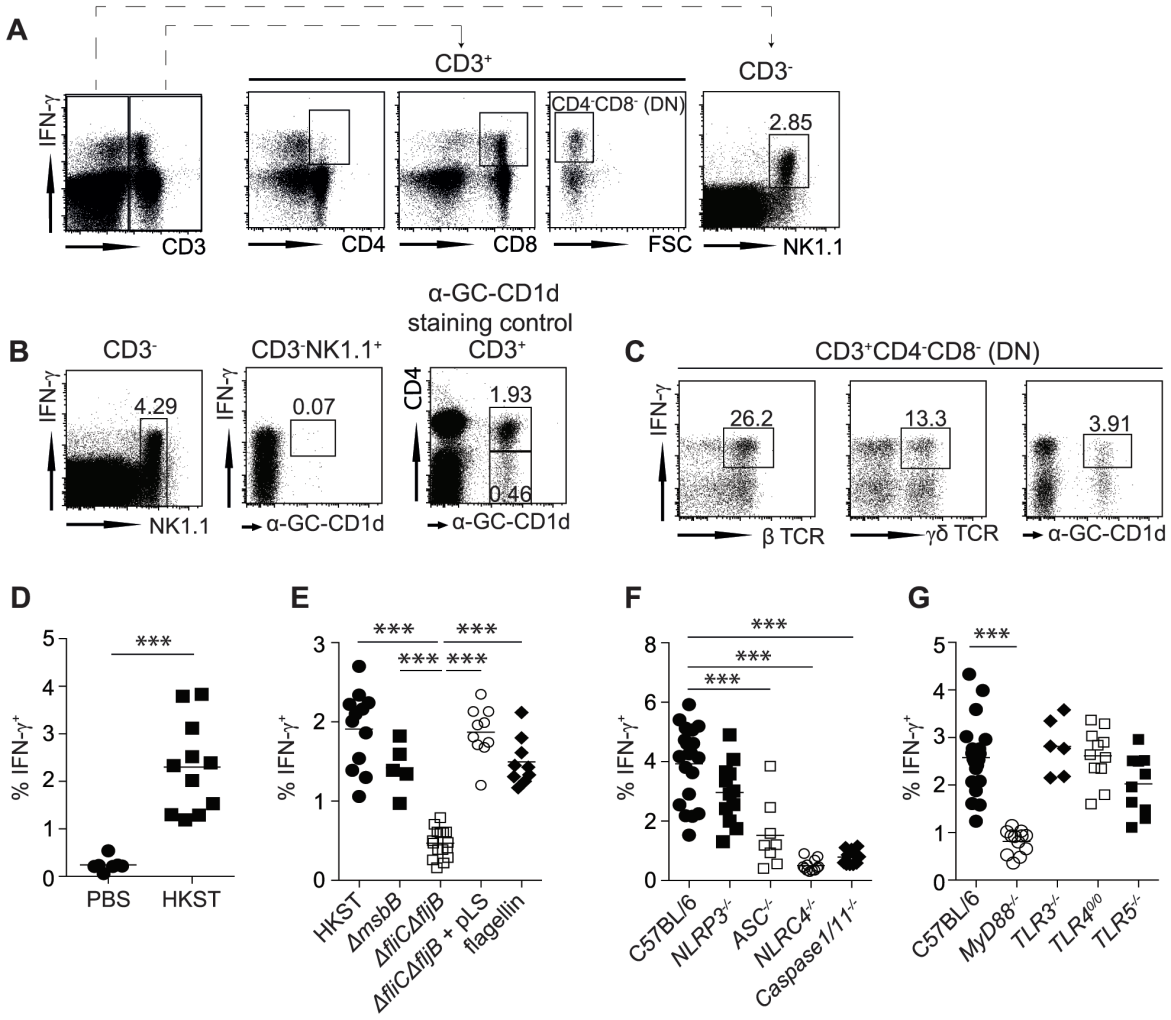
IFN-γ secretion by NK cells in response to *Salmonella* requires flagellin-sensing NLRC4 inflammasomes. (A–C) Naïve B6 mice were intravenously injected with 1×10^8^ cfu heat killed *S*. Typhimurium (HKST). IFN-γ secretion by lymphocyte subsets was assessed 2 h later in the spleen. Representative FACS plots of IFN-γ^+^ cells amongst CD3^+^ and CD3^−^ cells are shown (A). (B) CD3^−^NK1.1^+^ cells were assessed for the binding of α-GalCer-loaded CD1d-tetramers (middle plot) and compared to CD3^+^CD4^+^ and CD3^+^CD4^−^ cells (right plot). (C) CD3^+^CD4^−^CD8^−^ (DN) cells were assessed for IFN-γ secretion by β-TCR^+^, γδ-TCR^+^, α-GalCer-loaded CD1d-tetramer^+^ cells. (D–G) Percent of IFN-γ^+^ cells amongst total CD3^−^ cells of B6 (D, E), mice lacking key components in the inflammasome pathway (F), or TLR-signaling (G) 2 h after injection of 1×10^8^ cfu HKST (D, F, G) or *S*. Typhimurium mutants or 10 µg/mouse ultrapure flagellin (E). Individual data points (D–G) or representative FACS plots (A–C) from at least two independent experiments are shown. Statistical analyses: Paired Student's *t*-test (D), One-way ANOVA followed by Bonferroni multiple comparison test (E–G). *** p<0.001.

To determine whether cells other than memory CD8^+^ T cells innately secreted IFN-γ in response to *S*. Typhimurium, we exposed naive C57BL/6 (B6) mice to live or heat-killed *S*. Typhimurium (HKST) for 2 hours, as previously reported [3]. Apart from CD8^+^ T cells, CD3^−^ cells constituted the largest cell population that produced IFN-γ within 2 hours after *in vivo* exposure to HKST (Figure 1*A*). These IFN-γ-producing CD3^−^ cells expressed NK1.1 and did not bind to alpha-Galactosyl-Ceramide (α-GalCer)-loaded CD1d tetramers, which identified them as conventional NK cells (Figure 1*A, B*). Although IFN-γ secretion was also observed by a proportion of CD3^+^CD4^−^CD8^−^ (double negative; DN) T cells, these cells only constituted a very small fraction of total IFN-γ-producing cells (Figure 1*A*) and were comprised of a variety of different cell types, including γδ T cells and DN natural killer T (NKT) cells (Figure 1*C*). More conventional CD3^+^CD4^+^ T cells provided only a limited contribution to innate IFN-γ-production and the majority of these CD4^+^ cells were identified as NKT cells (not shown). Given that NK cells constituted the largest number of cells that rapidly produced IFN-γ in response to HKST, and therefore the largest population of potentially targetable cells, we subsequently investigated the possibility that despite their substantial inherent differences, innate NK cell- and memory CD8^+^ T cell-derived IFN-γ secretion were similarly regulated.

Having identified bacterial flagellin as the main driver of innate IFN-γ secretion by memory CD8^+^ T cells [3], we focused on the microbial stimuli that are required for IFN-γ secretion by NK cells. After exposure to whole HKST, approximately 2–3% of CD3^−^ lymphocytes in naïve B6 mice produced IFN-γ (Figure 1*D*), all of which were identified as being NK cells. When mice were instead exposed to HKST mutants that lacked a gene encoding a fully functional Lipid A of LPS (*ΔmsbB*) [7], or the major structural component of the bacterial flagellum (*ΔfliCΔfljB*), only the LPS mutant induced innate IFN-γ secretion (Figure 1*E*). These data suggested that flagellin was the main driver of innate IFN-γ secretion by NK cells. IFN-γ secretion by NK cells was reinstated by complementing the flagellin mutant strain with a plasmid expressing flagellin (*ΔfliCΔfljB* + pLS), and by injecting purified ultrapure flagellin (Figure 1*E*). Thus, analogous to antigen-independent IFN-γ secretion by memory CD8^+^ T cells [3], IFN-γ secretion by NK cells required the presence of bacterial flagellin.

### Inflammasome- but not TLR-signaling in CD11c^+^ cells is required for IFN-γ secretion

We have previously shown that innate IFN-γ secretion by memory CD8^+^ T cells required the presence of NLRC4 inflammasome components, such as caspase1, ASC and NLRC4 (see ref. [3]). These results were later substantiated by others, demonstrating a requirement for caspase1 in IFN-γ secretion by innate cells [5], [8]. We therefore reasoned that NLRC4-signaling was also important for IFN-γ secretion by NK cells in response to HKST. Indeed, two hours after exposure of *NLRC4^−/−^*, *ASC^−/−^* and *Caspase1/11^−/−^* mice to HKST, *ASC^−/−^* mice showed a significant (p<0.001) reduction and *NLRC4^−/−^* and *Caspase1/11^−/−^* mice a complete absence of innate IFN-γ secretion by NK cells (Figure 1*F*). In contrast, NK cells from *NLRP3^−/−^* mice secreted IFN-γ to a similar level as wild-type B6 mice suggesting a dispensable role for the NLRP3 inflammasome in initiating IFN-γ secretion by NK cells (Figure 1*F*). Considering that no differences in NK cell frequency between the investigated mouse strains were observed (data not shown), these results indicated that the induction of innate IFN-γ-release by NK cells also required the intracellular recognition of flagellin by NLRC4 inflammasomes.

**Figure 2:**
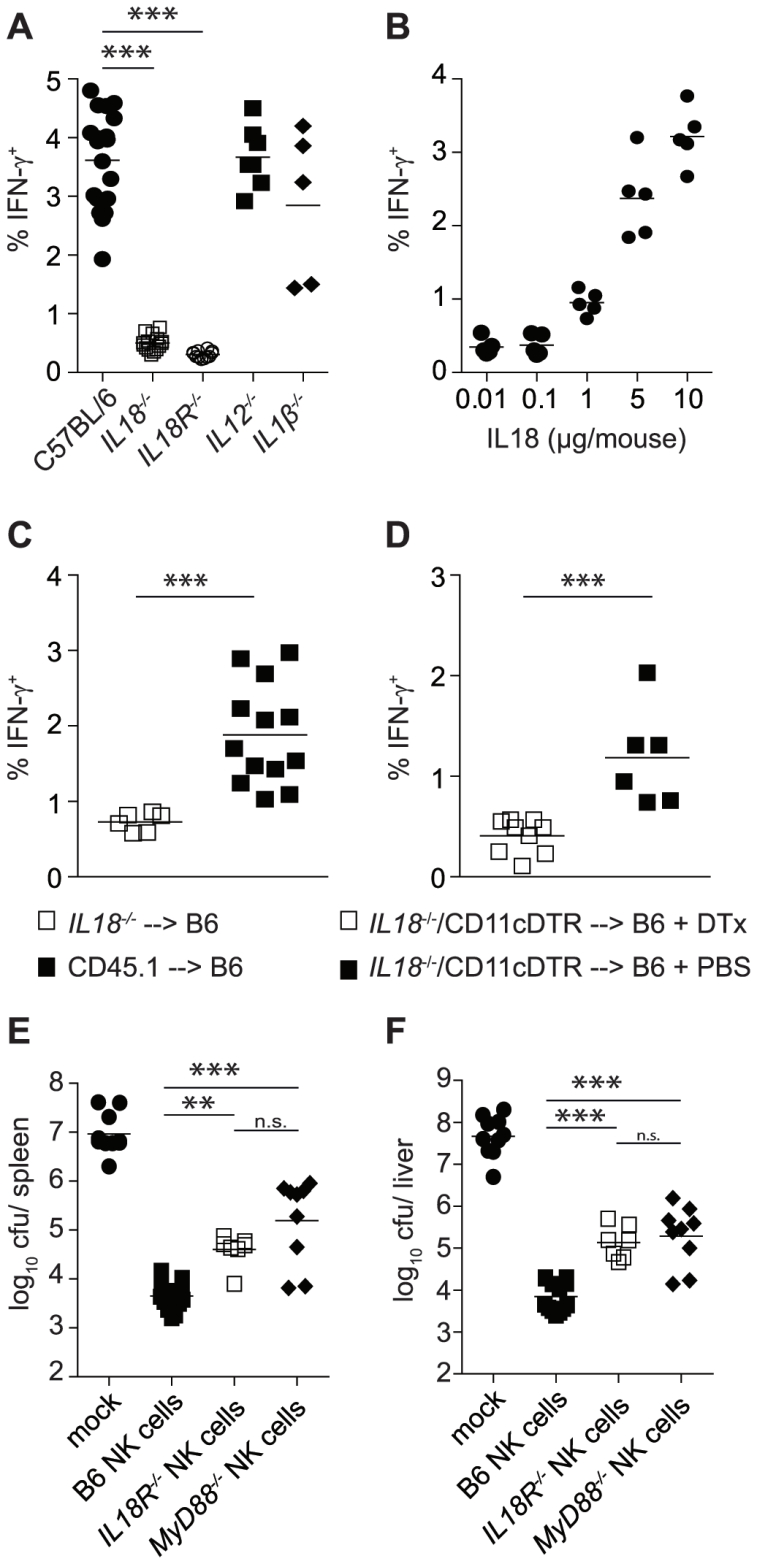
Dendritic cell produced IL-18 contributes to the production of host protective IFN-γ by NK cells. (A, B) Naïve B6, *IL18^−/−^*, *IL18R^−/−^*, *IL12^−/−^* and *IL1β^−/−^* mice were intravenously injected with 1×10^8^ cfu heat killed *S*. Typhimurium (HKST). Percent of IFN-γ^+^ cells amongst total CD3^−^ cells 2 h after injection of 1×10^8^ HKST (A) or different doses of recombinant IL-18 (B). (C, D) Percent of IFN-γ^+^ cells amongst total CD3^−^ cells straight bone marrow chimeras (C) or DTX-treated and PBS-treated mixed bone marrow chimeras (D) 2 h after injection of 1×10^8^ cfu HKST. (E, F) 1×10^6^ pure NK cells were intravenously transferred into naive *Rag2^−/−^γc^−/−^* mice on day 5 and 6 after culture. Mice were infected intravenously with 200 cfu BRD509 24 h later. Bacterial numbers in spleen (E) and liver (F) were assessed 23 days after infection. Individual pooled data points (A–D) of two (E, F) or three (A–D) independent experiments are shown. Statistical analyses: Paired Student's *t*-test (C, D), One-Way ANOVA followed by Bonferroni multiple comparison test (A, E, F) ***p<0.001, **p<0.01, *p<0.05.

To assess whether the flagellin-dependent secretion of IFN-γ by NK cells required the engagement of TLR-signaling, we exposed *TLR5^−/−^*, *TLR4^0/0^*, *TLR3^−/−^* and *MyD88^−/−^* mice, which all displayed similar frequencies of splenic NK cells, to HKST and assessed IFN-γ secretion two hours later. Similar to our observations with memory CD8^+^ T cells [3], of the TLRs tested, only the TLR-signaling molecule MyD88 was required for innate IFN-γ secretion by NK cells (Figure 1*G*). Consistent with MyD88 also being required for signaling of IL-18, the absence of IL-18 (*IL18^−/−^*) or its receptor (*IL18R^−/−^*) but not the absence of IL-12 (*IL12^−/−^*) or IL-1β (*IL1β^−/−^*) abrogated the ability of NK cells to secrete IFN-γ (Figure 2*A*). IFN-γ secretion by NK cells was induced by injecting purified recombinant IL-18 in a dose dependent manner (Figure 2*B*), and furthermore we have recently shown that serum IL18 levels after HKST injection are unchanged in the absence of MyD88 [3]. Combined, these results support a role for MyD88 in the induction of innate IFN-γ-release by NK cells at the ‘IL18Receptor-MyD88-axis’ but not in TLR-dependent NLRC4 inflammasome activation.

Finally, we further investigated the source of the NLRC4-dependent IL-18 release using straight and mixed bone marrow chimeras that were generated by injecting either *IL-18^−/−^* bone marrow cells alone (straight chimeras) or in a 1∶1 ratio with CD11c-DTR cells (mixed chimeras) into lethally irradiated B6 mice. After successful reconstitution of the immune system straight chimeras were injected with HKST. These experiments revealed that IL-18-driven IFN-γ secretion by NK cells was dependent on bone marrow derived, radiosensitive cells (Figure 2*C*). In order to determine if CD11c^+^ cells were the source of IL18, successfully reconstituted mixed chimeras were either mock-treated with PBS or injected with diphtheria toxin (Dtx) to deplete CD11c^+^ cells [9]. IL-18^−/−^/CD11cDTR mixed bone marrow chimeras treated with Dtx before administration of HKST demonstrated that innate IFN-γ secretion by NK cells only occurred when CD11c^+^ cells were capable of providing IL-18 (Figure 2*D*). Thus, analogous to the regulation of antigen-independent IFN-γ secretion by memory CD8^+^ T cells [3], IFN-γ secretion by NK cells required the provision of IL-18 to NK cells by CD11c^+^ cells. Collectively, these observations suggested a broad cooperation between inflammasome-mediated pathogen recognition and NK cell effector functions. To the best of our knowledge, this is the first demonstration that NLRC4 inflammasomes within dendritic cells are critically involved in IFN-γ secretion by NK cells, extending inflammasome-dependent NK cell effector functions beyond viral recognition through AIM2 inflammasomes [10].

### IL18-signaling-deficient NK cells only partially protect *Rag2^−/−^γc^−/−^* mice

The absence of IFN-γ producing NK cells after exposure of IL18-signaling deficient mice to HKST (Figure 1*G*, 2*A*) also directly translated into a lack of serum IFN-γ (data not shown). Given the importance of NK cell-derived IFN-γ in controlling *S*. Typhimurium-infections [4], we utilized our established NK cell transfer system into *Rag2^−/−^γc^−/−^* mice [4] to dissect whether IL-18-signaling was also crucial in mediating NK cell dependent host protection. We had previously shown that *Rag2^−/−^γc^−/−^* mice receiving wild type NK cells contained approximately 1000-fold fewer bacteria in spleen and liver 23 days after infection, compared to mock-treated animals, or mice that had received *IFN-γ^−/−^* NK cells [4]. Hence, if such NK cell-dependent host-protection was due to IL-18-mediated IFN-γ production, the transfer of NK cells (purity of transferred NK cells, assessed by expression of DX5 and NK1.1, was routinely above 98%) with defects in the IL-18 signaling pathway should lead to a similar impairment in the control of bacterial replication as the transfer of *IFN-γ^−/−^* NK cells [4]. However, although after transfer of IL-18R-deficient or MyD88-deficient NK cells approximately 10–50 fold more bacteria could be recovered compared to WT NK cell transfer (Figure 2*E, F*), overall these NK cells also yielded a significant (p<0.001) improvement in the control of *S*. Typhimurium infections and partially restored serum IFN-γ levels (data not shown). These findings demonstrate that inflammasome/IL-18-dependent IFN-γ production by NK cells is not the only pathway through which NK cells improve anti-*Salmonella* immunity *in vivo*. Clearly, redundancy in IL-18-independent mechanisms for IFN-γ production, provide important safeguard mechanisms against random loss of a single process.

These observations reported here imply that although the absence of individual pathways or effector mechanisms can have a significant impact, they often can be bypassed through alternative pathways and compensatory effects in infection studies in the whole animal. It appears that redundancy is a common phenomenon in infections with complex pathogens, such as *S. enterica*. Similar redundancy observations have been made when assessing virulence or immunological control using metabolic mutants of *Salmonella* [11] or host deficiencies in certain immune cell subsets [4], [12]. Although we do not know which IL-18-independent mechanisms contribute to NK-cell dependent host protection in our NK cell transfer model, the functional overlap between NK cells and memory CD8^+^ T cells reported here and previously [3], [4] could be of interest for therapeutic purposes. The role of inflammasome-dependent NK cell-derived IFN-γ might be exploited, for example, in settings where adaptive immune responses are absent or impaired, such as during severe *S*. Typhimurium infections in immunocompromised individuals [6], [13]. Given that both NK cells and memory CD8^+^ T cells rapidly proliferate and gain effector function in response to treatment with complexes consisting of IL-2/anti-IL-2 (see ref. [14]) and/or IL-15/IL-15 receptor α chain [15], it could be envisaged that cytokine complex treatment in conjunction with targeted inflammasome activation could serve as a potential novel approach to improve immunity against IFN-γ-dependent infections with *Salmonella enterica* and other intracellular pathogens. This could have significant implications for future vaccination and immunomodulatory strategies, in particular in immunocompromised hosts.

## Materials and Methods

### Ethics statement

All animal experiments were approved by The University of Melbourne Animal Ethics Committee (Permit Numbers: 06222, 0911513) and were conducted in accordance with the Prevention of Cruelty to Animals Act (1986) and the Australian National Health and Medical Research Council Code of Practice for the Care and Use of Animals for Scientific Purposes (1997).

### Mice

C57BL/6, CD45.1 (Ly5.1), *IL18* ^−/−^, *IL12* ^−/−^, *Rag* ^−/−^ *γc* ^−/−^, *TLR3^−/−^*, *TLR4^0/0^*, *caspase-1/11* ^−/−^ and transgenic CD11c DTR mice were bred and maintained at The University of Melbourne. *IL18R* ^−/−^ mice were kindly provided by W. Chen (Ludwig Institute of Cancer Research, Heidelberg, Australia). *ASC* ^−/−^, *NLRP3^−/−^*, *IL1β^−/−^* and *NLRC4* ^−/−^ mice were bred and maintained at the University of Lausanne and kindly provided by J. Tschopp (Department of Biochemistry, University of Lausanne, Epalinges, Switzerland). *TLR5^−/−^* mice were kindly provided by L. Alexopoulou (CIML, Marseille-Luminy, France).

### Bacterial strains and infection

*Salmonella enterica* serovar Typhimurium SL1344 (*S*. Typhimurium) were grown shaking overnight at 37°C in Luria Bertani broth (LB). *S*. Typhimurium Δ*msbB* were kindly provided by D. Maskell (Cambridge University, UK). For infections with *S*. Typhimurium BRD509 (a aromatic-dependent SL1344) bacteria were grown statically at 37°C in LB broth for 16–18 hrs, diluted in PBS and 200 cfu and injected into the lateral tail vein in a volume of 200 µl. The number of replicating bacteria was determined by homogenizing organs from infected mice and culture on LB agar plates supplemented with 25 µg/ml streptomycin.

### Assessment of *ex vivo* IFN-γ secretion

*Ex vivo* IFN-γ secretion by distinct lymphocyte subsets was assessed as previously described [3]. Briefly, mice were injected intravenously with 1×10^8^ cfu of heat inactivated *Salmonella* or purified flagellin (Invivogen, CA). Two hours after injection of bacteria the spleen was removed, single cell suspensions were prepared and red blood cells were lysed. 1×10^6^ cells were stained with the ‘Mouse IFN-γ secretion assay detection kit’ (Miltenyi Biotec, Germany) according to the manufacturer's instructions and IFN-γ secretion was analyzed by flow cytometry.

### Flow cytometry

To assess expression of surface antigens and IFN-γ secretion, viable, red blood cell-depleted single splenocytes were stained with monoclonal antibodies (BD Pharmingen) against CD4 (GK1.5), CD8α (53–6.7), CD3 (145–2C11), CD49b (DX5), NK1.1 (PK136), β-TCR (H57-597), γδ-TCR (GL3), IFN-γ detection antibody (Miltenyi Biotec, Germany) or α-GalCer loaded mouse CD1d tetramers (provided by D. I. Godfrey) as described elsewhere. After washing the cells, samples were analyzed using a FACSCantoII or LSRII analyzers (BD Biosciences, CA). Propidium iodide (2 µg/ml) was added to exclude dead cells.

### Isolation, enrichment and *in vitro* activation of NK cells

NK cells were negatively isolated from spleen and lymph nodes of donor mice using a NK cell enrichment kit and MACS technology (Miltenyi Biotec, Germany) according to the manufacturer's instructions. Isolated and purified NK cells were cultured with recombinant human IL-2 (Peprotech, NJ) as described [16]. Purity of cells after culture was determined by flow cytometry and 1×10^6^ NK cells were adoptively transferred intravenously on day 5 and 6 after culture into naïve Rag2^−/−^γc^−/−^ mice.

### Bone marrow chimeric mice and selective depletion of dendritic cells

B6 mice were lethally irradiated with 2×550 cGy and reconstituted with 5×10^6^ T-cell-depleted bone marrow cells from *IL-18* ^−/−^, transgenic CD11c DTR or CD45.1 mice. In some experiments irradiated mice were reconstituted with 1∶1 mixture of bone marrow from different mice. Chimeric mice were maintained on antibiotic water containing neomycin sulphate (25 mg/L) and polymyxin B sulphate (76900 U/L) for 6 weeks and were allowed to reconstitute for at least 8 weeks. Depletion of CD11c^+^ cells was achieved by injecting CD11c DTR chimeric mice intraperitoneally twice with 100 ng of diphtheria toxin (DTX) on day 3 and day 1 before the start of experiments. Effectiveness of depletion was routinely checked by flow cytometry.
